# Being right vs. getting it right: orientation to being recorded in psychotherapeutic interaction as disaffiliative vs. affiliative practice

**DOI:** 10.3389/fpsyg.2023.1254555

**Published:** 2023-11-22

**Authors:** Michael M. Franzen, Marie-Luise Alder, Florian Dreyer, Werner Köpp, Michael B. Buchholz

**Affiliations:** ^1^Institute for Media and Communication Studies, University of Mannheim, Mannheim, Germany; ^2^JUNKTIM Affiliated Institute of the International Psychoanalytic University Berlin, Berlin, Germany; ^3^Romance Linguistics, University of Freiburg, Freiburg, Germany; ^4^International Psychoanalytic University Berlin, Berlin, Germany

**Keywords:** conversation analysis, recording device, psychotherapy, reference, affiliation, disaffiliation, self-exploration

## Abstract

**Introduction:**

The study focuses on the orientation to being recorded in therapy sessions, emphasizing that these practices adapt to specific circumstances and influence subsequent actions. The study suggests a way to deal with the insolubility of the “observer paradox”: to accept that observation has an impact on the observed, but that the recorder is not necessarily a negative determinant. Furthermore, the study builds on the idea that participants' orientations to the recorder can be seen as actions.

**Methods:**

The data included in this study were collected from four psychodynamic therapies. A total of 472 sessions were searched for orientation to be recorded. Twenty-three passages were found and transcribed according to GAT2. Of the 23 transcripts, six excerpts have been analyzed as part of this article. The analysis of this study was done through Conversation Analysis.

**Results:**

The study explores how participants use the orientation to be recorded to initiate or alter actions within conversations, which can help achieve therapeutic goals, but can also hinder the emergence of a shared attentional space as the potential to disrupt the therapist-patient relationship. The study identifies both affiliative and disaffiliative practices, noting that managing orientation to be recorded in a retrospective design consistently leads to disruptive effects. Moreover, it highlights the difference between seeking epistemic authority (“being right”) and managing recording situations (“getting it right”) in therapeutic interactions as a means of initiating patients' self-exploration.

**Discussion:**

The integration of recordings into therapeutic studies faces challenges, but it's important to acknowledge positive and negative effects. Participants' awareness of recording technologies prompts the need for a theory of observation in therapeutic interactions that allows therapists to visualize intuitive practices, incorporate active contributions, counteract interpretive filtering effects, facilitate expert exchange, ensure quality assurance, and enhance the comprehensibility of therapeutic processes. These aspects outline significant variables that provide a starting point for therapists using recordings in therapeutic interactions.

## 1 Introduction: natural interaction involving being recorded

This article aims to further explore the areas of overlap between linguistics and psychotherapy-talk. This common area can be grounded anthropologically (Tomasello, 2014) insofar as human experience becomes describable in terms of publicly observable social actions rather than participant-interpreted internal mental activities. Accordingly, the present study attempts to focus on phenomena that themselves refer to the quality of observability and public accessibility.

Since Earl Zinn's first records of psychoanalytic therapies (cf. process research by Earl Zinn, 1933 in Kächele et al., 1973, p. 902), the therapeutic relationship as an observable process—or what was first coined by a patient of Sigmund Freud as “talking cure” (Freud and Breuer, 2004) and later called “therapy as conversation” (overview in Kurri and Wahlström, 2007, p. 315)—has been explicitly taken as a subject of scientific investigation (Friedman et al., 1978; Jaffe et al., 2001; Gulbrandsen et al., 2022). Crucial other developments such as the linguistic turn (Rorty, 2009), the relational turn in psychotherapy research (overview in Beebe and Lachmann, 2003, p. 379), as well as the replication crises in behavior research (Ioannidis, 2005), fostered the willingness to record therapeutic interactions, as well as to study these recordings methodologically.

In their study based on the recording of interactional occasions (Speer and Hutchby, 2003), the authors detect the problem of a “one-way mirror dilemma” (Speer and Hutchby, 2003, p. 333) “which treats [recorded interactions] […] as neutral mechanisms for the retrieval of information, as separate and distinct from the interactional and social contexts of which they form a part” (Speer and Hutchby, 2003, p. 334).

In other words, when observing a recording of an interactional process, you might think of a neutral position from which the interactional process is registered. However, it must be stated with Labov's observer paradox (Labov and Fanshel, 1977) that the more precise and differentiated an observation or registration process is carried out, the more the process becomes fixated (Bergmann, 1985). This, in turn, has the consequence that the observation itself intervenes in the process of being observed and changes it.

One way to deal with the insolubility of the observer paradox is to accept that observation has an influence on the observed, but “the presence of a tape recorder is not necessarily a determinate and negative force. Recording devices are not automatically significant and imposing, nor do they inevitably encourage only certain kinds of talk. […] [And] participants' displays of their awareness of the presence of recording technologies are not automatically a hindrance to interaction […]. Our point is that their reactions (whether positive or negative) can be analyzed as action” (Speer and Hutchby, 2003, S. 334). From the observer's perspective, participants' referring to the recording situation is particularly suitable for pointing out the necessity of a theory of observation or a communicative turn in therapeutic interactions (Buchholz et al., 2022).

## 2 Data and methods

### 2.1 Data and participants

We draw from four psychodynamic therapies with (i) 184 sessions in a modified psychoanalytic long-term therapy, two times a week, face-to-face, conducted and videotaped by an experienced male psychoanalyst, involving a male patient in his 40 s with a borderline diagnosis (PA3), (ii) 28 sessions of a fully audiographed psychoanalytic short-term therapy from the 1980s with a male patient in his 30 s with an obsessive–compulsive diagnosis (PA1) that has been researched extensively (see overview in Dittmann, 2016), (iii) about 180 sessions in another modified psychoanalytic long-term therapy with a male therapist and female patient (PA2), and (iv) 80 sessions in a depth psychological therapy^1^ with a male therapist and a female patient (DP1). Both therapies (PA2 and DP1) were diagnosed as depressive, and the therapies were conducted in the context of the Munich psychotherapy study (Huber and Klug, 2016). Patients gave their consent to audio recording in advance as part of the psychotherapy study.

### 2.2 Conversation analytic method

All 472 sessions were searched for orientation to be recorded. All 23 passages were found and transcribed according to GAT2 (Selting et al., 2011; Mondada, 2018). The authors analyzed all 23 transcripts using conversation analysis (CA), focusing on sequential organization and turn design. Out of the 23 transcripts, six excerpts have been analyzed as part of this study.

This study's analysis was conducted via CA. CA is a qualitative research method developed in the 1960s and 1970s by Harvey Sacks, Emanuel Schegloff, and Gail Jefferson (Sacks et al., 1974; Sacks, 1995 [1992]; Schegloff, 2007). It was originally intended to study the structure and organization of everyday social interaction. However, its scope quickly expanded to include the study of all types of spoken discourse, including institutional interactions, as psychotherapy (Pittenger, 1963; Schegloff, 1967; Lepper, 1996; Madill et al., 2001; Peräkylä, 2013, 2019; Buchholz et al., 2017; Horvath and Muntigl, 2018; Scarvaglieri, 2020). CA assumes that the dynamics of interaction depend heavily on the consistent orientation of the participants to the exchange and management of talk turns. Conversation analysts study this interactional behavior in great detail, how it reflects people's understanding of each other's actions in talk, and how social relations develop along with it. This analytic quality arises from CA's restraint perspective on participants' motivations. Instead, it focuses on observable utterances, patterns, and (in)regularities in multimodal shared interactions. By observing and analyzing the gradually unfolding conversation in interaction, conversation analysts then draw conclusions about how we establish and maintain connections with each other.

#### 2.2.1 Concept of affiliation

A fundamental concept in understanding how participants in a conversation support and endorse the storyteller's affective stance or treatment of the events being described is affiliation. It is distinct from alignment, which refers to the listener's support of the structural asymmetry in the storytelling (Stivers, 2008). Affiliation can involve the following two interrelated facets: (i) supporting the affective stance of the previous speaker and (ii) aligning with the action preference set in motion by the initiating action. Affiliative responses are maximally prosocial when they match the prior speaker's evaluative stance, show empathy, and cooperate with the preference of the prior action.

Actions that achieve affiliation in conversation include the use of verbal, prosodic, and visible resources to convey (dis)affiliation. For example, verbal resources such as adding intensifiers to evaluations can show strong agreement and affiliation. Response calls that do not distinguish between the speaker's and the respondent's feelings are affiliative. Verbal resources for affiliative reception, such as claims of understanding and congruent negative evaluations, can be accompanied by prosodic matching or upgrading. However, the same tokens can convey disaffiliation when delivered with prosodic downgrading.

Sequential positions also play a crucial role in the display of affiliation. Resources used to indicate affiliation in mid-flow positions, such as head nods, can be treated as disaffiliative at story completion. Instead, affiliative reception at story completion can be achieved with verbal resources like providing assessments or second stories. The role of sequential position in displaying affiliation is influenced by institutional settings as highly relevant for therapeutic interactions.

#### 2.2.2 Interrelations of affiliation and epistemics

Closely related to understanding how participants engage in conversation is the notion of epistemics. As previously discussed, affiliation in CA refers to the ways in which conversation participants establish connections. It involves various actions that signify shared understanding and cooperation. Epistemics in CA refers to how interlocutors display their knowledge, beliefs, or certainty.

Affiliation and epistemic interrelate in (i) sharing epistemic stances and expressing agreement and in (ii) conveying epistemic markers and affiliation strategies.

Firstly, participants often demonstrate affiliation by expressing similar epistemic stances. For example, when one speaker confidently presents information, and another participant responds in a way that supports this confidence, it demonstrates both affiliation and a shared approach to discussing the topic.

Conversely, participants may also show affiliation by expressing agreement or solidarity with each other's expressions of certainty or doubt. This highlights the nuanced interplay between epistemic positioning and affiliation as they contribute to the cooperative nature of the conversation.

Secondly, participants use various linguistic markers to convey their epistemic stance, such as hedging (e.g., “I think,” “maybe”), certainty markers (e.g., “definitely,” “certainly”), or modal verbs (e.g., “must,” “might”). These markers help signal their level of confidence or certainty in their statements.

Affiliation strategies, as discussed earlier, encompass actions like agreement, repair work, and preference organization. These strategies can be intertwined with epistemic markers to achieve affiliative goals. For example, agreeing with someone's statement may involve aligning not only with the content but also with the expressed level of certainty.

#### 2.2.3 CA contributions to the understanding of psychotherapeutic interactions with a focus on affiliation and epistemics

Certainly, CA has made significant contributions to our understanding of psychotherapeutic interactions, particularly concerning the concepts of (i) affiliation and (ii) epistemics.

In psychotherapeutic interactions, (i) affiliation has enhanced our insights into psychotherapy by at least three aspects: Firstly, CA studies have highlighted the central role of affiliation in building alignment between therapists and patients. Affiliation is manifested through various conversational actions, such as active listening, empathy, and agreement (Heritage and Maynard, 2006). Researchers have shown how therapists strategically employ affiliative responses, like cues (“mm-hmm”) and empathetic statements, to create a supportive and empathetic therapeutic environment (Heritage, 2011b; Buchholz et al., 2017; Stivers and Timmermans, 2020).

Secondly, CA research has demonstrated how therapists affiliate with the emotional stances of their patients. For instance, Stivers (2015) examined the use of affiliative responses in the context of emotional disclosures by patients. This research revealed that therapists often align with and validate the emotional experiences of their patients through affiliative actions, reinforcing the importance of empathy and understanding in psychotherapy.

Thirdly, managing resistance. CA studies have explored how therapists handle resistance and potentially disaffiliative behaviors from patients (Hutchby, 2002; Guxholli et al., 2007; Vehviläinen, 2008; Kent, 2012; Muntigl, 2013; Ekberg and LeCouteur, 2015; Bergen et al., 2018; Stivers and Timmermans, 2020; Fenner et al., 2022a,b). Peräkylä and Vehviläinen's (2003) research on resistance management in psychotherapy sessions showed that therapists employ affiliative strategies, such as paraphrasing and exploring patient perspectives, to address resistance without escalating conflicts. This nuanced approach helps maintain a positive therapeutic alliance.

Studies of (ii) epistemics in psychotherapeutic interactions have also advanced knowledge in at least three ways: Firstly, CA has highlighted the importance of therapists' epistemic stance in facilitating therapeutic conversations. Epistemic stance refers to how participants represent their knowledge, beliefs, or certainty about the information being discussed (Heritage and Raymond, 2005). Researchers have shown that therapists carefully modulate their epistemic stance, using markers such as hedging (“I think”) or certainty markers (“definitely”) to create a conducive environment for open dialogue (Heritage and Maynard, 2006).

Secondly, eliciting patient perspectives: CA studies have investigated how therapists employ epistemic actions, such as asking exploratory questions, to elicit patients' perspectives and experiences (Heritage, 2011a). This approach allows patients to actively participate in shaping the therapeutic discourse. It promotes a collaborative exploration of their concerns and narratives.

Shared epistemic stance: CA research has explored instances of shared epistemic stances between therapists and patients. By aligning their epistemic positions, therapists convey an understanding of and support for the patients' viewpoints (Muntigl et al., 2013). This shared epistemic stance fosters a sense of validation and trust, contributing to the effectiveness of psychotherapeutic interactions.

In summary, CA has made significant contributions to the understanding of psychotherapeutic interactions, primarily through the concepts of affiliation and epistemics. With regard to affiliation, CA research highlights its crucial role in psychotherapy, emphasizing how therapists strategically use affiliative responses, such as active listening and empathy, to establish rapport and a supportive therapeutic environment. In addition, CA has shed light on how therapists navigate emotional alignment and manage resistance using affiliative strategies, ultimately maintaining a positive therapeutic alliance.

In terms of epistemics, CA emphasizes the importance of therapists' careful modulation of their epistemic stance, using markers such as hedging and expressions of certainty to facilitate open dialogue. CA studies also explore how therapists use epistemic actions, such as asking exploratory questions, to elicit patient perspectives and encourage collaborative exploration of concerns and narratives. In addition, CA research highlights instances of shared epistemic stances between therapists and patients that foster validation and trust within psychotherapeutic interactions, ultimately enhancing their effectiveness.

## 3 Analysis and results

In the context of research on recorded therapeutic sessions, the following study examines participants' orientation to the fact of being recorded.

They do this by asking the respective other to focus on an object in the shared perceptual world, which is known in conversation analytic research as noticing (Schegloff, 2007; Muntigl and Horvath, 2014). Noticing implies references to events, actions or objects/persons. But not every reference is a noticing. In noticing the previous perception is verbalized, which gives reason for verbalization, and is thus made part of the interaction. That means regarding the recording device: If the patient notices that the recording is running, a device is standing there, the device is new, etc., then it is a noticing. However, if the device is referred to in the course of an argumentation or it is asked why the recording is being made, etc., the action is different, for example, an account, a question, and a request.

Referring to the recording device differs from noticing. Although reference is made to an object in the perceptual environment of both participants, it is not made as a reference to the physical object but as a transformed “object of conversation” (Buchholz, 2016), which is in line with more recent models of reference: It is not the extra-linguistic world *per se* that is mapped or referred to, but rather “the mental concepts we make of the world” (Brinker et al., 2000, p. 306). In this context, discourse is a resource for conveying and shaping the mental constructs participants hold about their environment, highlighting the interplay between language, perception, and cognition in interaction.

### 3.1 Orientation to the recording device as *affiliation*

We assume that “observable orientations to the fact of being recorded […] have a range of interactional uses and relevancies for the current interaction as it unfolds in real time” (Speer and Hutchby, 2003, S. 325) and found two different routes or practices of orienting to the fact of being recorded.

The analyzed data indicate an overall division of the phenomena into those that are oriented toward the recording device and perform therapeutic goals through different actions, for example, that patients explore themselves or elaborate on the previously referred recording device. “In many therapies, it is a central principle that patients should examine their own experiences” (Vehviläinen, 2008, S. 123–124); in short, they are affiliative. Secondly, there are those sequences that do not reach that effect, for example, when patients do not explore themselves or elaborate on the previously referred recording device; in short, they are disaffiliative (see ch. 4.2).

#### 3.1.1 Extract 1: DP1 2nd session

In this first example, it will be shown how the participants orient to the recording device of a therapy dyad in the 6th min of a second-depth psychological psychotherapy session between a male therapist and a female patient. In the beginning, the patient claims that she does not know what to say today because she might be in such a good mood. The therapist offers to understand this as a fear of losing her good mood by discussing it with the therapist. The patient confirms this and says that she has been feeling bad for a long time. However, she likes to tackle problems directly. Seemingly abruptly, the therapist refers to the recording device.

##### 3.1.1.1 Extract 1.1, minute 5


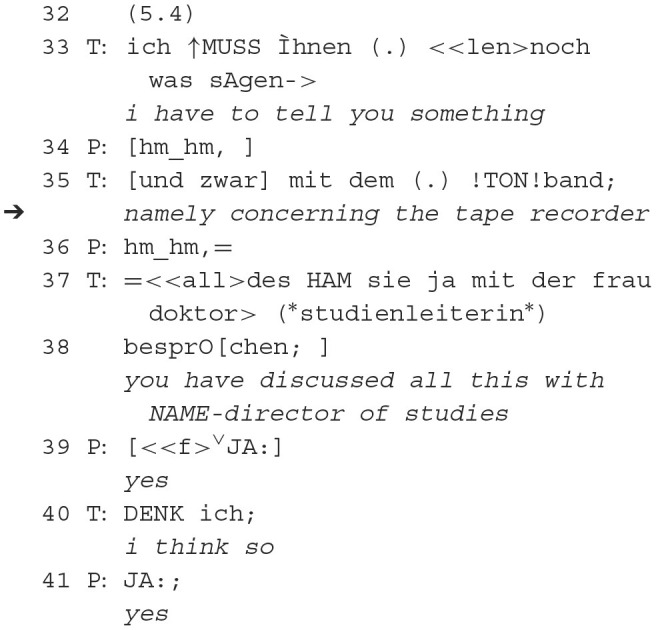


In the first place, the remark about the tape recorder is positioned as a reference for follow-up actions by the therapist but also as an expression of authority requiring consent (39, 41). In order to make the following sequence relevant as one that requires active patient participation, the therapist states his knowledge of what he thinks what the patient should already know (37–38). He wraps his knowledge in a polar question (37–38). This entails agreement with the patient, albeit overlapping and loudly intoned. The therapist initiates the patient's agreement by a subsequent hedge (40), which marks uncertainty. The power of the polar question does not extend beyond the next turn, and the reference to the “tape recorder” (35) is answered by the patient as an attempt to project follow-up actions of a polar question (yes/no answers), similar to how anticipatory objection treatment has already been described in therapeutic contexts as “getting to yes” (Muntigl et al., 2020).

In summary, in this sequence, the therapist places an announcement (“I have to tell you something”, 33) in order to prepare the **reference to the recorder** (35) that is secured via a polar question (37–38) and finally confirmed by the patient. The patient aligns with the communicative project (Clark, 1996) of the therapist by answering the polar question in a progressive way and thereby confirming the initiating announcement of the therapist. The preannouncement of the therapist conveys that he will now be addressing a delicate subject. Not only delicate to the patient but probably delicate to himself.

Although the sequence becomes clear as an affiliative one, the therapist marks his epistemic stance by means of hedging (40). This uncertainty marker is not explicitly responded to; thus, we will look at the proceeding of the sequence in order to pursue a further explication of the epistemic dimension and, thus, better understand the function of the reference to the recording device.

##### 3.1.1.2 Extract 1.2, minute 6


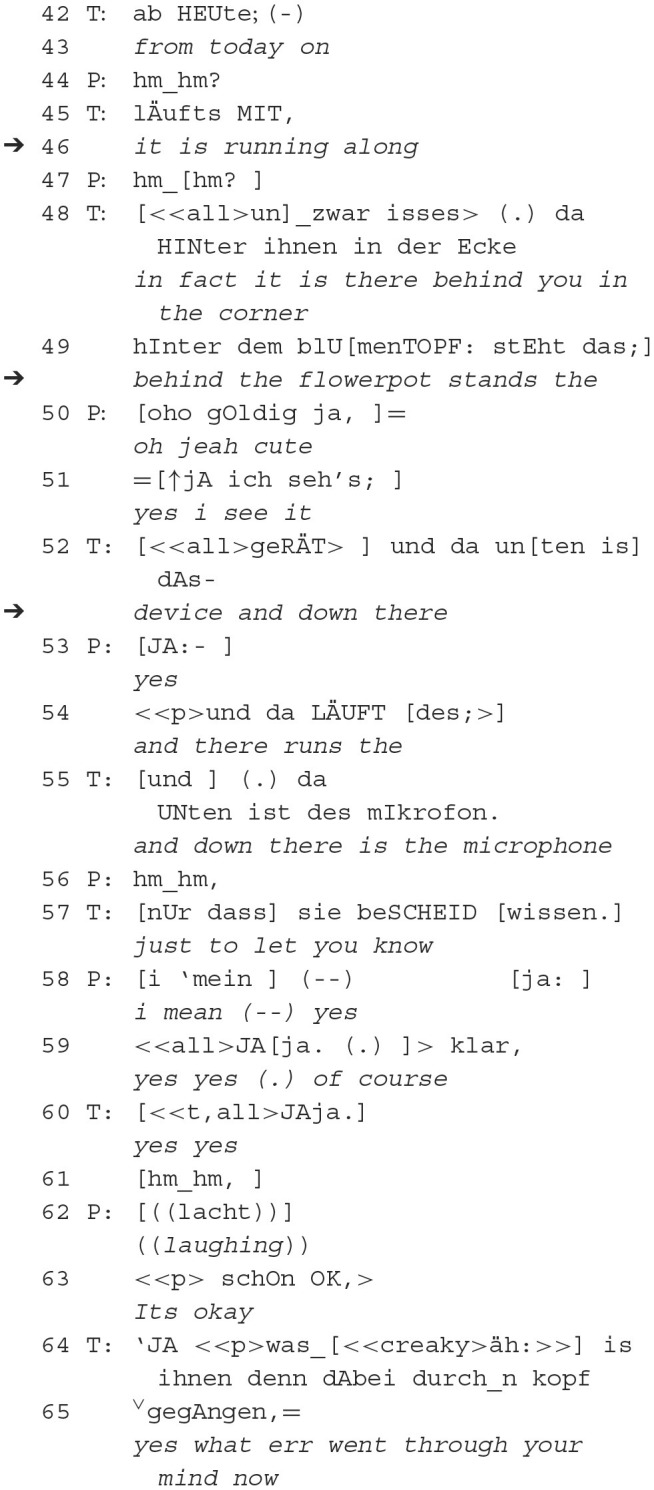


In this second continuation of the first sequence, one conversational problem arises, namely, what further actions are relevant to the interlocutors? The orientation to being recorded by the therapist does not seem to entail any clear follow-up actions on the part of the patient, who places slightly questioningly intoned continuers (43, 45). Thereby aligning and continuing with the therapist's project to explore the recording device and its position in the room, but also conveying disaffiliation, not clearly supporting the affective stance of the therapist.

The reference to objects in the perceptual environment of both participants, “behind the flowerpot there” (47), initiates the patient's overlapping subsequent utterance, first an ironic attribution of “cute” (48) and then another overlapping placed acknowledgment of the perception, which can also be called “to attend to” (s. German “aufmerken” in Brinkmann, 2016). Noticing establishes the attentional space as shared (Tomasello and Rakoczy, 2003; cf. joint attention in Tomasello and Rakoczy, 2003). Together, the two actions form a shared activity (Clark, 1996) of attending to/noticing and *being* attended to/confirming perception. This conversational reference sequence is made relevant again by the therapist: “and down there the microphone runs” (53). It is uttered as a postponed second utterance part in order to attenuate the shared attention space and possible subsequent utterances by means of epistemic downgrading “just to let you know” (55) after a patient's continuer (54). This is further demonstrated by the reciprocal closure efforts of the participants (56–61). An elaboration prompt by the therapist (62, 63) defines preferred subsequent actions, namely the recording device or microphone as an occasion for self-exploration or associative subsequent utterances.

In the context of the second and the first affiliative example, the conversational problem of follow-up actions becomes clearer: It appears that the therapist treats the reference of the camera as a delicate issue [“i have to tell you something” (33) and “I think so” (40)]. The presence of the videotape does not challenge the patient here. However, it rather constitutes a potential impingement on the therapist's epistemic status. Due to the fact that the agreement to the recording of the therapy is done beforehand with the study's director, the therapist has to, on the one hand, secure this patient as part of the study (confirmation to being recorded is necessary to be part in the study) and on the other hand, he has to create a working relationship, building on trust in him as the therapist. That dilemma is being solved by not only the therapist but also the patient. They create an affiliative sequence of referencing the recorder. The ironic utterance “cute” by the patient can also be heard as a comment toward the therapist's effort to hide the audiotape behind the flowerpot. Subsequently, she reassures him that “it's okay” (63). Finally, the therapist refocuses her attention on his uncertainty by reaching out for relevant therapeutic actions enabling change, namely the self-exploration of the patient.

#### 3.1.2 Extract 2: PA1 5th session, minute 35

In the 36th min of the 5th h of a brief modified psychoanalytic therapy of a male therapist and male patient, the therapist points out the initiative competence of the patient by calling him a “fiercely determined young man”. Subsequently, the patient laughs, and the therapist interprets this action as a “vehemence” transformation. The patient elaborates on this interpretation as a “puzzle” (Vehviläinen, 2008), which is the pre-sequence to the following excerpt:


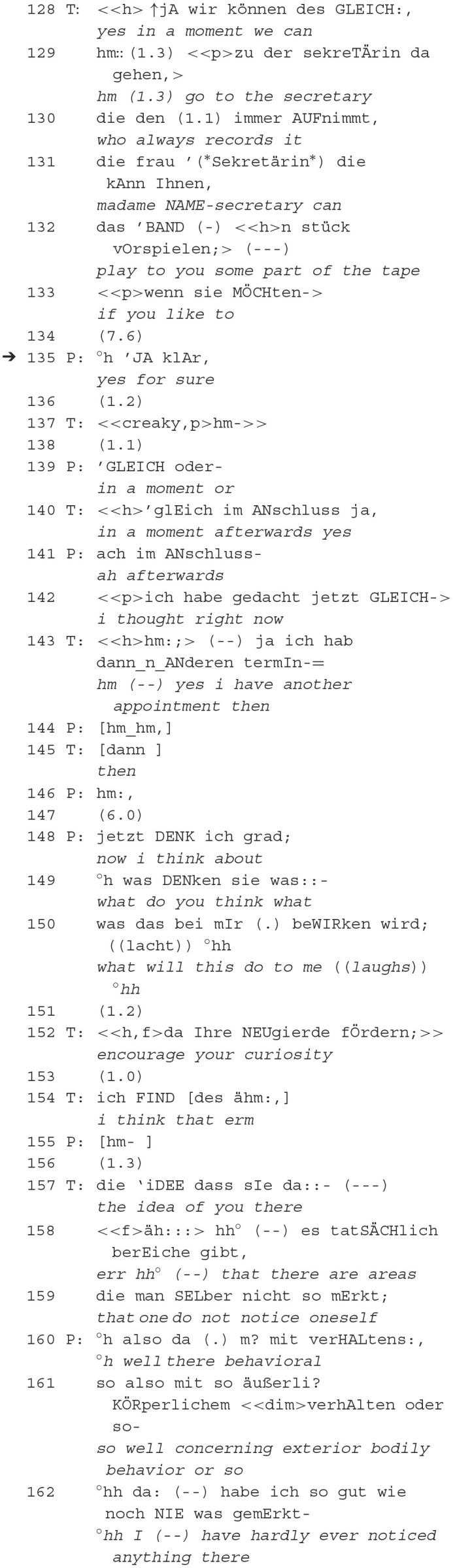


The therapist places a reference to the “tape” (132) and explicitly links possible subsequent actions: “go to the secretary” (129). After a longer pause (134), the patient agrees to the suggestion. When asked by the patient, the therapist specifies that “in a moment afterward” (140) is meant instead of here and now and that he accompanies the patient but will not be part of the follow-up action himself (143). Like in the previous excerpt, the reference to the recording device is explicated by the therapist. After the self-disclosure or explication of the therapeutic intention (152, 157–159), the patient connects that communicatively competent with a mental issue (161, 162), which was not a topic before. The therapist's initiatives of (i) watching the video recording alone after the session (132) and (ii) disclosing the intention of expressing this suggestion (152) can also be understood as offering solutions to the patient's formulations of the problem (123–124 and 148–150). In such a way that “the patient has to find himself in the mind of the clinician […] to experience a mind being changed by a mind” (Bateman and Fonagy, 2016, p. 182). This implies that the therapist takes the patient as the primary actor who can see by himself certain (behavioral) evidence of (certain) psychological issues. And this is certainly a socializing strategy into practices of self-observation and reflexivity. Additionally, the therapist indicates the replay of the video after the session. At the same time, he sees other patients, which would help the patient continue the work beyond the encounter with his therapist.

In summary, the therapist initiates a proposal to go to the secretary (128–129), then attaches the relevant follow-up action again as a proposal via **orientation to being recorded**: to watch the tape of today's session (130–132). The explicit linking of the orientation to being recorded, plus the affiliative co-creation of the summons-answer sequence, led to the patient's initiation of self-exploration (160f). Affiliation and epistemics are closely linked in this context. Firstly, they are intertwined because gaining access to an epistemic status that is not yet known or balancing epistemic status by explaining future actions can be considered prerequisites for establishing affiliation. This is achieved by aligning one's actions with the preference set by the initial action. Secondly, the summons-answer sequence creates opportunities for affiliative interactions based on shared epistemic perspectives. This can involve acknowledging mutual understanding and agreeing on evaluations, forming a basis for shared epistemic stances.

#### 3.1.3 Extract 3: PA2 1st session, minute 19

In this psychoanalytic therapy, we zoom in on the first session at minute 19 with a male therapist and a female patient. Prior to this excerpt, the therapist insinuates that it might be him who hinders her from speaking freely, even though the patient might think of him as her analyst to whom she should be able to talk. The patient imagines “only a tape recorder” as a recipient, claiming the problem of not being able to speak freely lies in her and has nothing to do with the therapist. After self-interrupting her further thoughts about the recording device, she explicitly asks the therapist whether he understands, which makes the answer relevant.


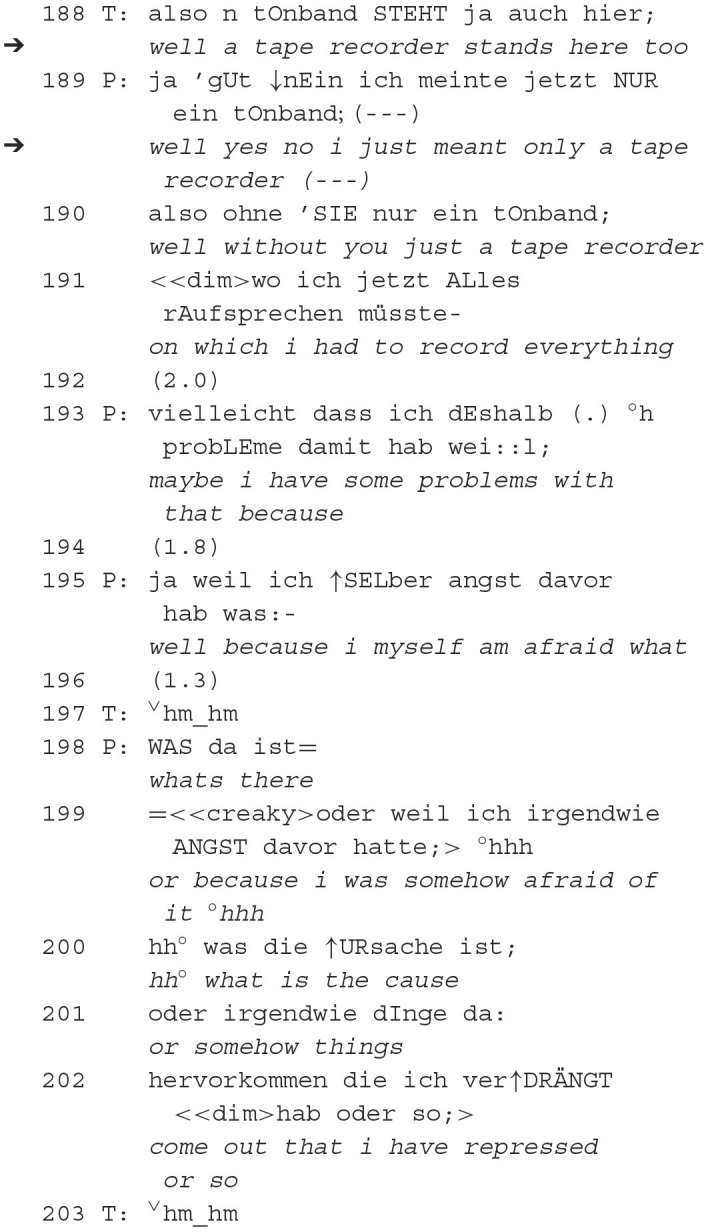


The therapist complies with this request to speak only after a long pause and initiates a repair of the patient. Interestingly, the repair is initiated by means of an orientation to being recorded located in the local environment, which, however, is not “determined” as noticing, i.e., made identifiable (e.g., by certain articles, description of the place, or actually pointing to it). The misunderstanding (Hinnenkamp, 1998) is already indicated by the long pause (187) after the patient's request for understanding. It is cleared up by the patient, who refers to a fantasized situation: “without you—just a tape recorder” (190). In this case, and unlike in excerpt 1.2, the therapist does not enable the subsequent actions of self-exploration by a direct request. The therapist, like in extract 2, responds to the patient's request to speak, thereby initiating the patient's work on redressing the epistemic balance. The patient elaborates that she is “afraid” (199) that “things […] will come out that I have repressed” (201–202). With the help of the mental experiment (only recording device, no resonant therapist), she addresses the problem previously mentioned by the therapist, according to which he is not only a receiver (165) but someone listening.

In summary, in this excerpt, the **recording device** is initially referenced as a physical object in the local environment by the therapist [“a tape recorder stands here too” (188)], which serves as a reminder of an epistemic balance. However, the recording device is then transformed by the patient into a conversational object [“without you—just a tape recorder” (190)], as the patient uses the recording device as a starting point for her self-exploration. Interestingly, the patient aligns with the therapist by the action preference set in motion by his initiating action—this (at least transformation into) orientation to the recording device differs from the following examples.

### 3.2 Contrast examples: orientation to the recording device as *disaffiliation*

In contrast to the previous excerpts (therapeutic goals via orientation to being recorded) in the following transcripts, the orientation to the recording device is recognizable as *disaffiliation*, which serves to address delicate content. These often “retrospective orientations to the inappropriateness of taping certain topics […] tend to be […] bound up with activities of teasing or complaint-making. What we find here is a form of situated morality in which participants use the presence of the recording device to establish that what has just been said is problematic in some way” (Speer and Hutchby, 2003, S. 325). Interestingly, in order to implement the retrospectively organized sequences of orientation to the recording device as evidence or “being right,” the interlocutor's following disaffiliative actions are “a vehicle for getting someone to do something; […] telling someone that you know better is equivalent to telling them what to do” (Antaki, 2012, p. 544).

#### 3.2.1 Extract 4: PA2 109th session, minute 33

In this extract from the session that we have already become familiar with (see last extract 3), the patient makes a disaffiliative, incongruent evaluation. She objects to the therapist's statement about her being not warm-hearted. The therapist gives an example of her being warm-hearted with her partner in order to demonstrate her misunderstanding. However, the two do not reach a shared epistemic status. The patient continued to talk about her irritation about what she understood. The therapist doesn't object. Instead, he addresses the recording of the last session:


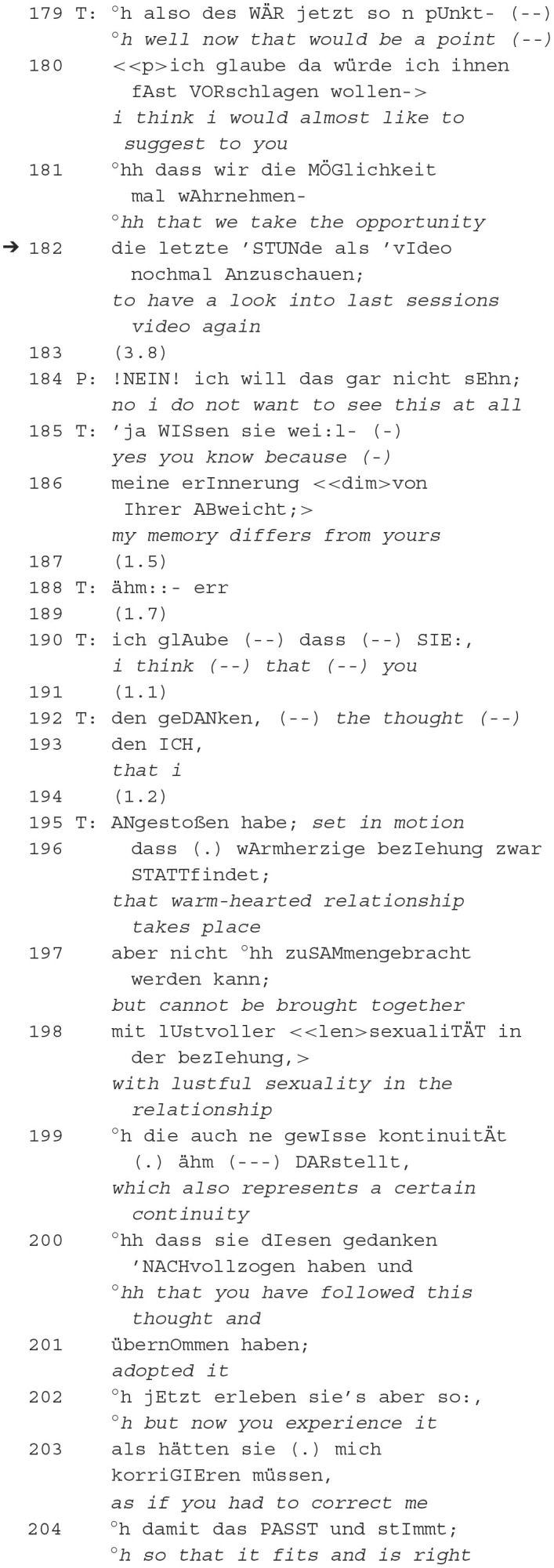


Like in the previous extract 3, a misunderstanding is triggered via the therapist's utterance, according to which the patient is warm-hearted in the relationship but cannot live a “lustful sexuality” (198). The patient, however, understands that she is not warm-hearted. The therapist shows the expected dispreference of his following utterance by placing relativizations in the epistemic domain (179–181: “would be,” “i think,” “almost like to suggest,” “take the opportunity”), conveying a rather downgraded epistemic stance, and asks her to “have a look into last sessions video” (182). After a longer pause, the patient clearly refuses (184). The therapist justifies his request by referring to the different memories (186) and introduces watching the recording as a resource for clarifying the indecision. However, watching the recording is not realized further on, insofar as the orientation to being recorded fails as a request to initiate a joint project “clarifying misunderstandings”. No possible follow-up actions are made explicit by the therapist (unlike in PA1 extract 2), which indicates the long pause after the therapist's suggestion (183) as a possible search for implications. The patient's rejection of the action proposal to view the recording of the previous meeting is followed by a justification for the recording by the therapist in the third position. Thus, the therapist makes an unsuccessful attempt here to present a difference between himself and the patient as in need of repair—for which the previously placed proposal for action was made relevant as a solution. The focus on the proposed action is related to a subsequent action. Thus, to be realized, “viewing the recording of the last hour” is proposed as a common solution.

The misunderstanding seems to have been clarified, and the therapist is probably more interested in being right and showing the patient that she presented the misunderstanding as if he should have corrected it. One could speculate that the therapist wants the conflict to be resolved for him, but this could be the starting point for the patient to reflect on her misperception and her response to the therapist's comments. Clinically formulated, this could be a revealing of unconscious acting. However, the therapist shifts the topic from the sexual relationship in the patient's partnership to the therapeutic relationship in the here and now—and the video serves to “clarify” her behavior or to make it accessible to her self-exploration. Therefore, the video serves to observe the performative level of the patient's and therapist's actions (Deppermann et al., 2020) in order to make these invisible insights viewable and hearable.

This results in opposing courses of action in the epistemic domain: (i) Retrospectively through the thematization of different memories (“clarification of misunderstanding”), whereby what was previously said can potentially be given a new meaning. (ii) Projectively through the design of a potentially subsequent action “watching the recording of the last hour”. In this respect, the orientation to being recorded is placed in a projective sequence. However, only after the patient's rejection the sequence is extended but not taken up by the patient.

In summary, the therapist embeds the **reference to the recorder** in a request to replay the video (180–182). Even though he anticipates the other's dispreferred answer, he leaves out an explicit reason. The patient rejects the request (184). The therapist treats the rejection as OIR and formulates an account for his request (185–186). The patient stays silent after her rejection—what is sequentially understandable as silencing (Thiesmeyer, 2003; Dimitrijević and Buchholz, 2021). The therapist then continues formulating the patient's potential thoughts (191ff) but does not evoke an answer. All in all, it becomes clear that the unresolved misunderstanding affects not only the epistemic domain in terms of different epistemic statuses but also the level of cooperation as disaffiliative. The orientation to being recorded as a resource fails. Even a further self-exploration of the patient does not take place. The sequence illustrates how the orientation to being recorded silences the patient's subsequent utterances, which is also evident in the fact that after the patient's contradiction (“look into last sessions video” 182), no further utterance signals are placed by her. Using the recording as evidence for therapist's reasoning or as a resource to convict the patient does not seem to be effective in furthering the pragmatic therapeutic goal of patient's self-exploration and results in the reference to the recorder as disaffiliation.

#### 3.2.2 Extract 5: PA3 22nd session, minute 09

In this interaction between a male dyad in a modified long-term psychoanalysis in their 22nd session in minute 9, the therapist initiates by proposing a shared project and offers his perspective on what has been discussed so far. The patient initially accepts this offer but later seeks clarification. Eventually, the patient uses the opportunity to introduce his perspective, leading to a mismatch in conversational projects as the therapist attempts to redirect the conversation. This exchange demonstrates a misalignment in the conversational projects of the therapist and the patient within the psychoanalytic context. The different projects are being explicated in the following excerpt.


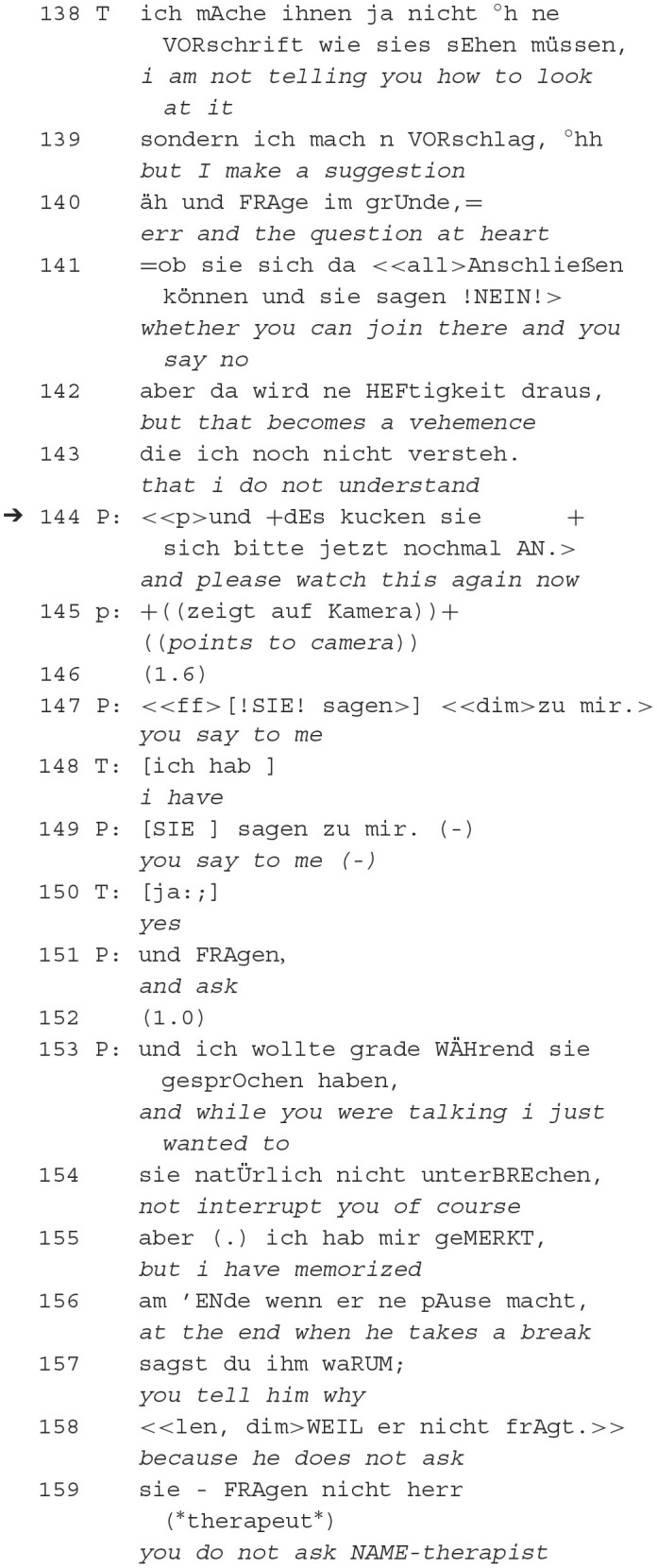


After the indirect rejection of the therapist's request, the patient places an argumentative reference (Sacks, 1995 [1992])—by pointing to the video recorder and asking him to watch the recording (144). Subsequently, the patient explicates that he has signaled to the therapist's listenership in order not to interrupt him (155), nurturing the plan to formulate a reproach. He accuses the therapist of telling the patient something instead of asking (159). Only after the patient's orientation to being recorded (144–145), the interactional history is rearranged (153) from a claim of listening into a reproach (159).

The request to focus on the recording refers to a reference potentially perceptible to both. The patient uses the reference as a resource to reinterpret the interaction history in the conflict context (as in PA2 extract 4) and to place potentially delicate utterances in the conversation. This form of retrospective reference enables the connection to the previous interactional history in such a way that a new meaning is produced. The retrospective negative reevaluation of putative listener signals calls into question mutual trust in the participants' activities as reliable and positively intended. From a psychotherapy research perspective, this is a possible alliance rupture that presses for repair as therapy progresses.

In summary, in this sequence, the patient initially demonstrates affiliation by providing listener signals (line 155) that indicate active listening. These signals are essential for maintaining the flow of the conversation and ensuring a supportive environment or affiliation. As the conversation progresses, the patient interprets the therapist's actions and places a reproach (line 159) that reinterprets his benign display of affiliation into a hostile calculation. This shift from signaling active listening to reproach illustrates how affiliation can be challenged within a conversation. The patient's reproach implies a breakdown in the therapist-patient affiliation, which is crucial for a productive therapeutic interaction.

Also, in terms of epistemics, the patient introduces an argumentative reference by mentioning the recorder (line 144). This reference functions as evidence to support the patient's perspective, reflecting his epistemic stance. The patient seeks to establish a basis for his argument by invoking the potential evidence provided by the recorder. The patient engages in retrospective reinterpretation (lines 153 and onwards) of the interaction history, using the reference to the recorder as a resource for disaffiliation. This reinterpretation involves attributing new meanings to past actions and statements, emphasizing the role of epistemics in shaping the patient's understanding of the therapy session. The negative reevaluation of putative listener signals calls into question mutual trust in the therapeutic relationship. This aspect highlights how epistemic elements, such as trust and reliability, play a crucial role in the therapeutic context. The mention of a potential alliance rupture underscores the significance of maintaining a positive epistemic stance to ensure effective therapy (Safran et al., 2001).

The patient's use of retrospective reference and argumentation serves to assert his epistemic position while influencing the affiliative dynamics of the interaction, ultimately shaping the course of the therapy session.

#### 3.2.3 Extract 6: PA3 29th session, minute 15

The following sequence is of the same dyad as the previous one. In the 29th h, in minute 15, the therapist initially attempts to build affiliation by suggesting that they should reflect on something together. He acknowledges the patient's way of speaking, demonstrating attentiveness and an attempt to align with the patient's communication style. The therapist then shares his observation with the patient about the latter. This itself is a delicate issue because it entails other attributions. He suggests that sometimes, probably contrary to the patient's intention, he may express himself in a very devaluing way. This reflects an epistemic stance, where the therapist judges the patient's communication. He points out the patient's perception and the need to take it seriously, emphasizing the epistemic dimension of understanding the patient's viewpoint. The therapist further addresses the patient's critics of the therapist's lack of empathy and lack of authenticity. Moreover, he expresses uncertainty about the sincerity of the patient's devaluating utterances. The therapist works to balance affiliative and epistemic dimensions of the therapeutic relationship. He fosters understanding and collaboration while addressing potential issues in the therapeutic relationship. While the patient clears his throat, the therapist concludes:


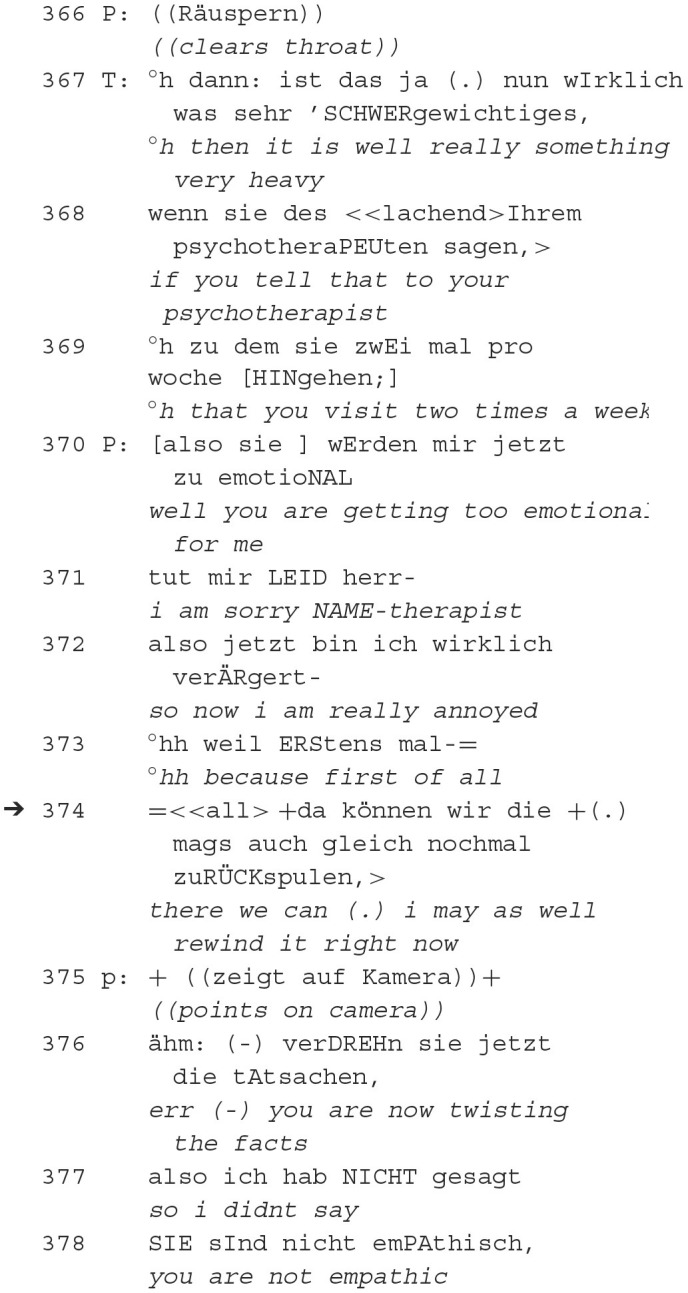


After this intensification of the conflict, the patient interprets the therapist (as “too emotional”, 370), places an ironic preface (371) and then leads over to his emotional mood (“annoyed”, 372) before using the recording device as a resource for the placement of a reproach (376) by means of argumentative reference.

That this is a misunderstanding (regarding what the patient remembers having said and what the therapist says he heard from the patient), which is commented on by the patient (361) and not the content of the therapist's confrontation (341–42), becomes clear retrospectively, as the patient says: “i didn't say you are not empathic” (377–78).

However, as in the previous excerpt of the same dyad (PA3 extract 5), the patient's reference to the interaction history of the patient's utterance (361) becomes retrospectively the first part of a reproach sequence—with the current sequence as the second part (376).

The confrontational moment (as in the previous excerpt), in which the therapist questions the alliance with the patient, again serves as a starting point for the patient to introduce the recording device as an argumentative counter-weapon. This thus placed orientation to being recorded indicates that it has a fundamental quality of authentication—it functions like evidence in court and thus, in turn, reflexively indicates the severity of the rupture.

In summary, the patient interprets the therapist as “too emotional” emphasizing his epistemic stance (evaluative judgment). The patient then uses the recording device as an argumentative reference, highlighting the role of epistemics in supporting his perspective and shifting the interaction's dynamics. Retrospectively, it becomes clear that there was a misunderstanding, emphasizing the importance of shared epistemic understanding for affiliation. The confrontational moment becomes a starting point for the patient to introduce the recording device as evidence, reflecting its authentication function. Overall, this interaction illustrates how affiliation and epistemics interplay in shaping the therapeutic discourse and dealing with conflicts and misunderstandings (s. also extract 5).

## 4 Discussion: how does the orientation of being recorded differ for affiliative vs. disaffiliative practices?

The orientation to being recorded reveals a set of practices that are “context-sensitive, as routinized uses of resources for situated actions that are flexibly adapted to the specific circumstances in each case” (Selting, 2016). These practices evoke subsequent actions accordingly; they introduce elements from perception what is available to both participants in the communicative space. The common ground can be both—confused or stabilized.

As illustrated above, the practices of orientation to being recorded involve actions taken by participants in response to the presence of a recording device during the therapy session. These practices serve specific functions within the conversation: (i) In some cases (extracts 1–3), participants use orientation to be recorded as a way to initiate a subsequent action. For example, the presence of the recording device might prompt the therapist or patient to bring up a particular topic or issue for discussion. (ii) In other instances (extracts 4–6), participants use this orientation to alter the meaning of what has already been said. This retrospective transformation suggests that the recording device can influence how participants interpret and frame their previous statements.

The study identifies proactive sequences, which are initiated by both the therapist and the patient, as well as retrospectively organized sequences, which are opened exclusively by the patient. Retrospective sequences often involve bringing up something that was previously withheld or not fully addressed.

This study hints at the idea that certain conversational content when retrospectively introduced can potentially disrupt the common ground between the therapist and patient. This suggests an epistemic dynamic where the act of retrospectively bringing up certain issues can destabilize the therapeutic relationship. Therapists may need to be sensitive to these dynamics and address any imbalances or feelings of discomfort that arise when such issues are revisited in therapy. These instances reveal the interpretation of who is the one who listens and observes. It demonstrates that not only the therapist scrutinizes the patient's behavior. The patient, too, examines the therapist carefully. Nothing new to clinicians. But it once more shows how patients are themselves competent interactants. They use the evidence of the recording device to gain sovereignty of interpretation in a possible unbalanced power dyad.

The six therapeutic interaction sequences related to the orientation to being recorded differ in their effects regarding affiliation (Muntigl et al., 2012; Muntigl and Bänninger-Huber, 2016):

Excerpts 1–3 refer to effects that are affiliative or related to the therapeutic goals, for example, patient self-exploration; excerpts 4–6 show disaffiliative effects, for example, reproachful actions by both participants. It is clear from the data in this study that managing orientation to be recorded in a retrospective reference design (excerpts 4–6) always leads to a disaffiliative effect. This confirms findings from a previous study of participants' orientations to being recorded in mundane conversations (Speer and Hutchby, 2003).

The role of the communicative environment is crucial for the interpretation of how therapists interpret patients' (disaffiliative) actions, i.e., when “psychotherapists became stressed when their patients did not want to apply suggested techniques, but rather withdrew, thus jeopardizing the therapeutic alliance. Psychotherapists interpreted this as an expression of the disturbed thought world of their patients, who were then addressed with the same techniques” (Buchholz and Kächele, 2019 transl. by MMF). The result is a rupture. The danger is that if this rupture is not repaired, “repeated cycles” (Castonguay et al., 1996) might emerge.

As a clinician, keeping this “sensitizing concept” (Blumer, 1954) in mind when recording the therapeutic work could prevent potential disaffiliative rupture cycles. If the therapist plans to use the recording device, it is also important to inform and discuss the recording with the patient before the recorded sessions begin. There are study contexts in which patients have to confirm the recordings in order to participate in the study. However, privacy laws usually declare the right to withdraw from the recording of sessions. So, even if patients may not be able to continue therapy in the study context, it is also true that they can at least decide ex negativo about the recordings.

When analyzing the sequences, it is noticeable that in three excerpts with disaffiliative effects, video replay as evidence is used (extracts 4–6). In the case of disaffiliative effects, one could note an expression of a dissonance reduction strategy, namely “being right”, to be the holder of epistemic authority. While therapists have deontic authority only (Stevanovic, 2011; Stevanovic and Peräkylä, 2012; Ekberg and LeCouteur, 2015), including the duty to give reasons for requests (unlike extract 6). Instead of being right, what allows speakers to refer to being recorded is when they “get it right”. That is, to manage orientations to being recorded as a resource, namely through actions, such as explicating follow-up questions (e.g., extract 3) or embedding them in an if-formulation (extract 4). On the other hand, disaffiliative practices are not synonymous with poor therapy outcomes. To encounter resistance in therapy does not mean to fail, but to work on the resistance with the idea of enabling the patient to a self-repairing process—one day, without the therapist.

The orientation to being recorded can be a resource, as is especially evident in the follow-up actions that realize a common therapeutic goal (see excerpts 1–3). However, there are equally contrasting examples of how a shared attention space fails to emerge (see excerpts 4–6).

By referencing the nature of the situation in which they are recorded as one that can be viewed in a temporally displaced manner, a method becomes visible with the help of which participants establish the situation as a public and observable one—and observe one another being recorded.

### 4.1 Why record therapies? “There are areas that you do not notice yourself” (extract 2)

Finally, to return to the beginning of the article, the integration of recordings into the study of therapeutic interactions is challenged by Labov's observer paradox, whereby detailed observations may inadvertently fixate and thereby influence and alter the observed process. However, it is important to recognize that the presence of recording devices is not inherently negative or deterministic. Participants' awareness of recording technologies can either facilitate or hinder interactions, and these reactions can be analyzed as actions (Speer and Hutchby, 2003, p. 334). From an observer's perspective, participants' references to the recording situation highlight the need for a theory of observation or a communicative turn in therapeutic interactions (Buchholz et al., 2022):

i) **Visualization of intuitive practices:** Within the perspective on the communicative performance of therapeutic work, it becomes possible for therapists to learn to link their own intuition to the observations of the recording, in order to make, for example, therapeutic interpretive strategies visible. Empirical studies, for example, on interpretations in psychotherapeutic sessions offer some suggestions (Peräkylä, 2004, 2010, 2011).
ii) **Participants' active contribution:** This allows them to take a new position to the formerly intuitive interpretive practice. It helps to be sensitized for future contexts. Recordings can be used to identify and record interpretive strategies and their relationship to therapeutic theories. This also includes that patients and therapists both actively contribute to it instead of starting from Freud's mirror metaphor, which assigned therapists a neutral position, i.e., uninfluenced by the interaction (Thomä, 1974).
iii) **Interpretive filtering effect** (Druckman et al., 2009): Recordings are an option for therapists to counteract a conflict of interest between attention to note-taking and listening. Taking notes then filters possible interpretations of the here and now. As the recording can then be used as a reference point, the therapist's attention can be fully focused on the therapy. Verbatim dialogue, transcriptions or videos can then be used as the basis for a summary for therapeutic, legal, and billing purposes or for scientific evaluation (Kächele et al., 1973).
iv) **Exchange between experts:** Intervision groups of therapists and conversation researchers can help to work on one's own blind spots since it is only in the course of the transcript or video analysis that known memories or conceptions are combined in such a way that the epistemological gap between theory and practice can be bridged by generating new conceptions. In this context, it is important to mention the institutionalization of practice and research by JUNKTIM e.V. An association founded in 2020 for empirical conversation research in psychotherapeutic interaction (Franzen and Alder, 2023).
v) **Documentation as quality assurance instrument:** The “data secure and facilitate the way back to the latent thoughts which, according to theory, must become conscious on the part of the patient in the course of the process and, as far as they concern the countertransference, should be at least partially conscious, i.e., formulable. […] They can […] trace the processes […] back to a rather faithful starting basis which can be restored at any time. That manifold evaluations thus obtain a secure basis is indisputable” (Kächele et al., 1973).
vi) **Comprehensibility:** Documentation of therapeutic hours can provide a basis for research and further development on an empirical basis by opening up therapeutic processes to the outside and making them comprehensible. The records are “a prerequisite for the clarification of certain psychotherapeutic and psychoanalytic questions […] [which] make[s] possible that not only the two directly involved in the therapeutic process give information, but also third parties can deal with the material” (Kächele et al., 1973).

These six points do not claim to be complete but essentially map the influencing variables that follow Labov's observation paradox and mark a starting point for therapists working with recordings of therapeutic interactions.

### 4.2 Future research

Most of the recorded orientations thus contribute as a resource to the establishment of a shared attentional space. However, some passages were also found to represent disaffiliating actions and a potential rupture for the therapeutic work. How to deal with these aspects in a therapeutic way has already been described elsewhere (Safran et al., 2001). However, in relation to the recording situation, it could be the subject of future research. Multimodal forms of practices of orientation to being recorded could also be included in further research activities. It could be interesting to see how a longitudinal study of recording orientations could find different individual local management in a dyad or group case in the context of a series of instances and their changes or routines over time. Another line of interest might be to explore how the patient's (self-)observations are reflected.

## Data availability statement

The raw data supporting the conclusions of this article will be made available by the authors, without undue reservation.

## Ethics statement

Written informed consent was obtained from the individual(s) for the publication of any potentially identifiable images or data included in this article.

## Author contributions

MF: Conceptualization, Data curation, Formal analysis, Funding acquisition, Investigation, Methodology, Project administration, Resources, Software, Supervision, Validation, Visualization, Writing – original draft, Writing – review & editing. M-LA: Formal analysis, Methodology, Supervision, Validation, Writing – review & editing. FD: Formal analysis, Methodology, Supervision, Validation, Writing – review & editing. WK: Data curation, Supervision, Validation, Writing – review & editing. MB: Formal analysis, Funding acquisition, Resources, Supervision, Validation, Writing – review & editing.

## Footnotes

^1^The concept of “depth psychology-based psychotherapy” is unique to Germany. The setting is a one-week face-to-face session of 50 min. Originally born out of the need for a “smaller psychoanalysis” financed by the health insurance companies, an independent form of therapy soon emerged. Usually, up to 100 therapy hours are paid by the health insurance (for psychoanalysis, up to 300 h). Depth psychology therapy revolves around addressing unconscious conflicts and pathological psychological issues within the therapeutic relationship. However, it is more focused than psychoanalysis, which uses free association. Additionally, therapists may employ either more active or more reserved intervention techniques, as described by Jaeggi and Riegels (2018), Wöller and Kruse (2014), Rudolf (2019), and Hauten (2021).
