# Supplementary figures and images for: Being right vs. getting it right: orientation to being recorded in psychotherapeutic interaction as disaffiliative vs. affiliative practice
Source: Front Psychol. 2023 Nov 22;14:1254555. doi: 10.3389/fpsyg.2023.1254555 (PMC10704604; doi:10.3389/fpsyg.2023.1254555)

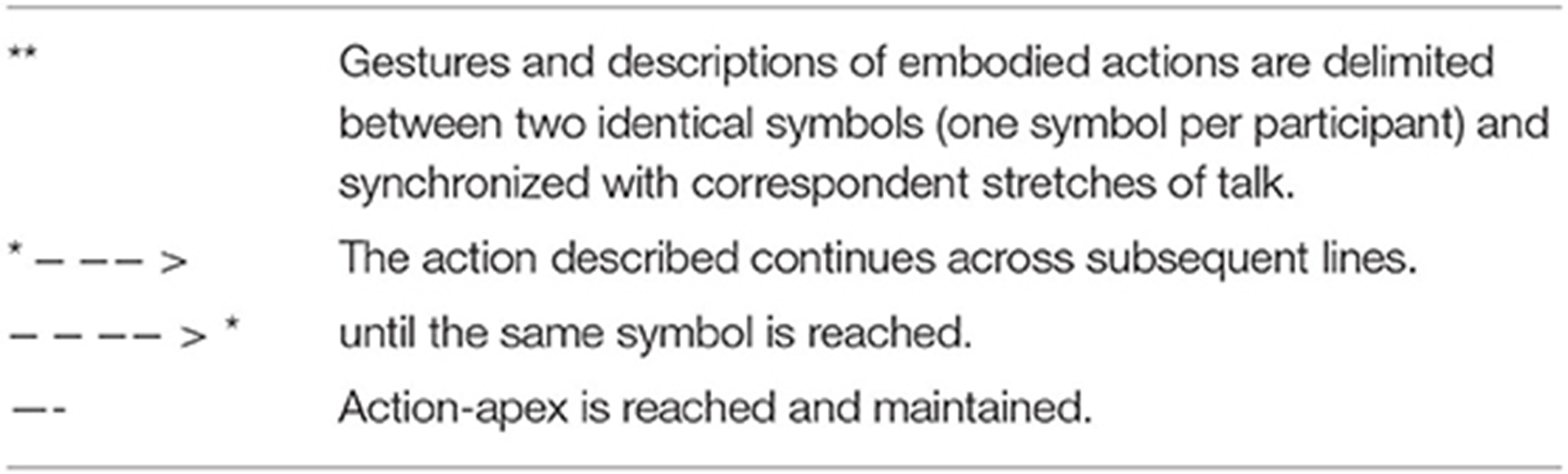

Supplement: Supplementary file 1 [file Image_1.JPEG]

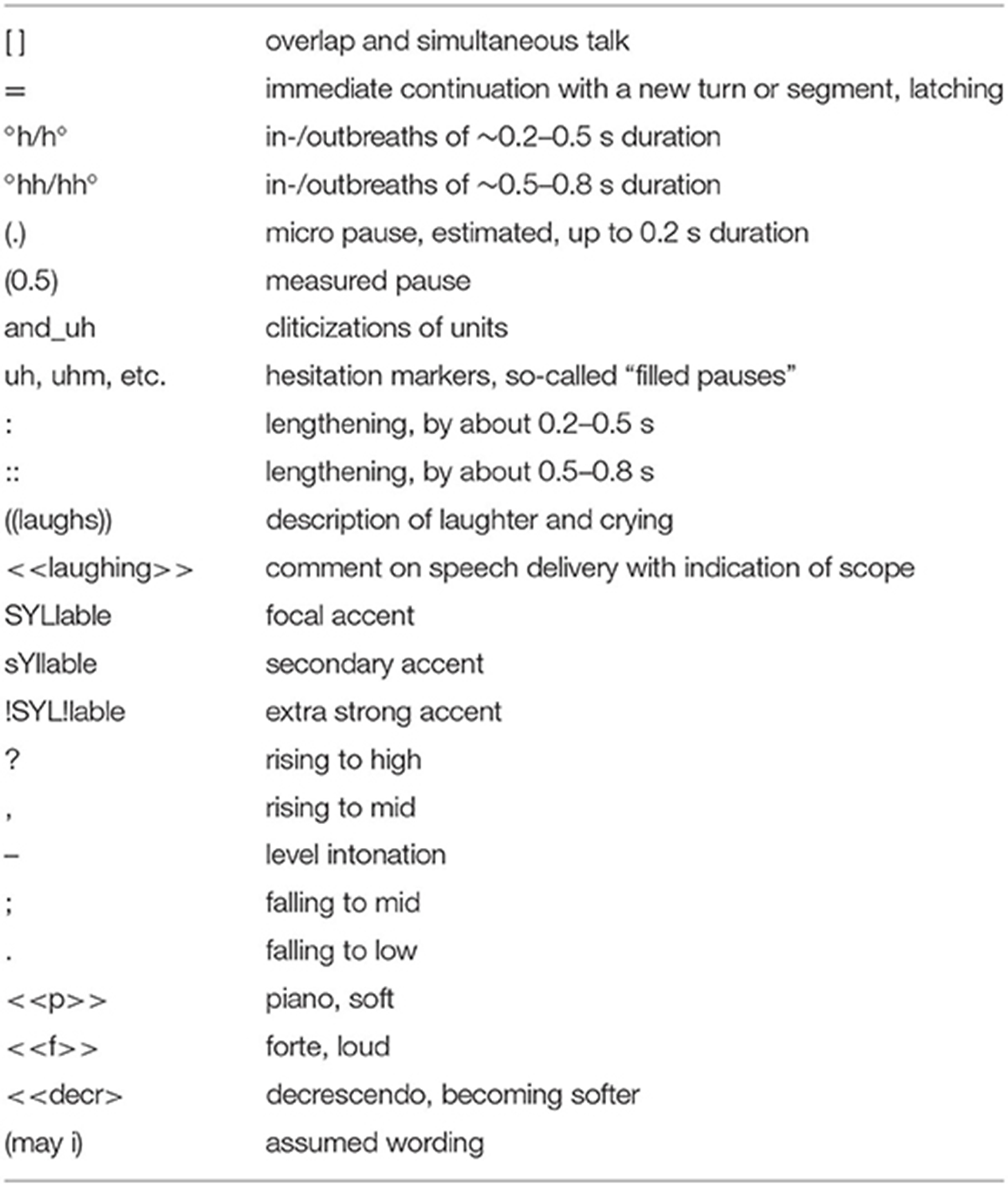

Supplement: Supplementary file 2 [file Image_2.JPEG]
